# Hadra’s Operation for Cystocele

**Published:** 1887-09

**Authors:** F. H. Tucker

**Affiliations:** San Augustine, Texas


### HADRA'S OPERATION FOR CYSTOCELE—OPERATION, CURE.

*By F. H. Tucker, M. D., San Augustine, Texas.*

For Daniel's Texas Medical Journal.

ON the 8th of July I performed the following operation, which I shall please to call "Hadra's operation for cystocele." The patient 35 years of age, mother of three children. Not having an appliance for securing patient, I had a leather belt made to encircle waist of patient, one ring made fast to belt on each side. I then flexed the legs on the thighs, and the thighs on the abdomen, passed a belt around the leg just below the knee, crossed it between leg and thigh, crossed again over the thigh, passed the ends through the rings in the waist belt and drew them tightly and secured all well. I could then bring buttocks of patient to edge of the table, either on the side or back.

A crescentic transverse incision was then made on the anterior lip about three-fourths of an inch off from the os, with the finger and handle of scalpel separated the vaginal wall from the tissues, lifted the flaps up for about 1¾ inches; with a strong tenaculum passed through the rent. I hooked the cervix and brought it well downwards and forward; then with an anneurismal needle I passed a whip-cord silk ligature through the cervix, tied the ends together, and gave to an assistant to make traction; a continuous suture was transversely carried on the highest point of the flap, thus tacking the vagina to the cervix. Bringing the edges of the wound together did not seem to raise the cervix quite high enough. I cut away a narrow strip from the upper flap. I then brought the edges of the wound together, and secured them by another continuous suture.

The operation thus completed, I washed out the vagina with a bi-chloride solution 1-4000 strength, dusted the parts with iodoform, packed around the cervix with absorbent cotton, and put the patient to bed.

I am inclined to think in all cases where there is a superabundance of tissue in the vagina it would be good practice to re-sect a transverse portion of the vaginal flap, as was done in this case. It at once lifts the cervix up nearer a natural position, and flattens the cystocele more.

The patient is now comfortable, going around the premises with no support but the vaginal packing of absorbent cotton. The final result in this case promises to be perfectly satisfactory. With a little care to avoid wounding the ureters and bladder, the operation is one of comparatively easy execution.

